# I-131 remnant ablation after thyroidectomy induced hepatotoxicity in a case of thyroid cancer

**DOI:** 10.1186/s12876-015-0281-7

**Published:** 2015-05-07

**Authors:** Rong Lin, Omar Banafea, Jin Ye

**Affiliations:** Department of Gastroenterology, Union Hospital, Tongji Medical College, Huazhong University of Science and Technology, Wuhan, 430022 China

**Keywords:** Hepatotoxicity, Drug-induced liver injury, I-131, Thyroid cancer

## Abstract

**Background:**

Radioactive iodine (I-131) is routinely used for the treatment of differentiated thyroid cancer following surgery. Drug-induced liver injury (DILI) is a leading cause of acute liver failure. Here we reported a rare case of diffuse hepatic uptake (DHU) of radioactive iodine (I-131) induced hepatotoxicity in patient with I-131 ablation therapy after thyroidectomy.

**Case presentation:**

A 57-year-old woman was admitted due to jaundice, itching and dark urine with abnormally elevated liver function. She has performed thyroidectomy followed by 100mci radioactive I-131 ablation therapy 21 days ago. The basic hepatic protection could not efficiently prevent disease progression. The patient was further treated with methylprednisolone, the bilirubin and alanin aminotransferase were finally lowered back to normal in the follow-up visit.

**Conclusion:**

To the best of our knowledge, this is the rare description of DILI complications in thyroidectomy patient due to I-131 ablation therapy. The patient responds to glucocorticoid therapy well, but not basic hepatic protection treatment. Even though this is only a single case, it reminds physicians make DILI in early consideration when patient present liver injury after I-131 ablation therapy.

## Background

In recent years, it has become common to administer radioactive I-131 as part of the therapeutic strategies in patients with thyroid carcinoma following the surgical procedure of total or partial thyroidectomy [1]. Ideally, I131 treatment should only ablate residual micro/macroscopic tumor cells in the thyroid and/or distant metastasis but not affect healthy tissues in the body. However, the posttherapy scans frequently reveal diffuse I-131 uptake in the liver [2-5]. This does not represent metastases in the liver, which appear as discrete lesions. Drug-induced liver injury (DILI), the term that describes abnormalities in liver function tests related to medication intake, is a leading cause of acute liver failure [6]. However, whether the diffuse hepatic uptake of I-131 following I131 ablation therapy would induce DILI remains unclear in thyroid cancer patient with thyroidectomy.

Here we report a rare case of DILI complications in thyroidectomy patient due to I-131 ablation therapy. There are three reasons make our case particularly all the more worthy of attention. First, the radioactive I131 ablation is a routine therapy for thyroid cancer patient after thyroidectomy; Second, I-131 ablation therapy could present diffuse hepatic uptake; Third, DILI is the leading reason for acute liver failure; I131 induced DILI does not response to the basic hepatic protection.

## Case presentation

**Figure 1:**
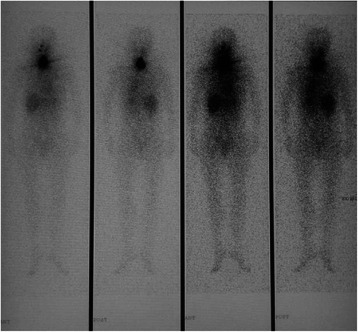
Emission Computed Tomography (ECT) 5 days after I^131^ treatment. As shown, I^131^ were uptaken mainly in the thyroid bed, but also in the liver, stomach, oral and nasal cavity.

A 57-year-old Chinese woman was admitted to our unit with a one-week history of jaundice, itching and dark urine. She denied history of liver disease, metabolic syndrome (obesity, diabetes, hypertension, dyslipidemia) and alcohol exposure. She has been diagnosed as thyroid cancer (papillary thyroid cancer) and performed total thyroidectomy 3 months ago. The patient was further treated with 100 mCi radioactive I-131 therapy 21 days ago (Emission Computed Tomography (ECT) image taken 5 days after I131 therapy was shown as Figure 1). Before starting I131 treatment, she had undergone ultrasonography of the liver and serum biochemical tests including liver chemistry, with all results being within the normal range.

**Figure 2:**
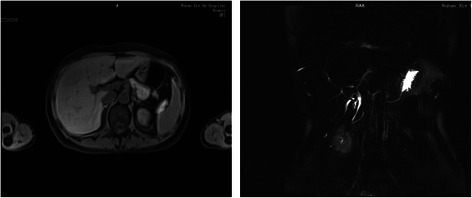
Enhanced Magnetic Resonance imaging(MRI) and Magnetic Resonance Cholangio Pancreatography (MRCP) of this patient were normal.

On examination she presented with skin scratching, yellowish discoloration of the skin and sclerae (eyes), no edema, ascites nor encephalopathy were presented. Laboratory tests were abnormal: elevated total bilirubin (TB) = 131.1 μmol/L (7 folds above the upper limit of normal); Direct bilirubin(DB) = 82.1 μmol/L (10 folds increased); alanin aminotransferase(ALT) = 370 μ/L (9 folds increased); Aspartateaminotransferase(AST) = 189 μ/L (4.5 folds increased); gamma-glutamyltransferase (GGT) = 275 μ/L (6 folds increased); alkalinephosphatase (ALP), serum albumin, total bile acid and international normalized ratio (INR) were normal. Viral hepatitis (HAV(Ab), HBV (Ag/Ab), HCV(Ab, RNA),HEV(Ab), EBV(Ab), CMV(Ab) and HSV(Ab)), autoimmune serologies (antinuclear antibodies, anti-smooth muscle antibody, anti-mitochondrial antibody/M2, anti-liver-kidney microsomal antibody, anti-liver cell plasma antibody, anti-soluble liver antigen/liver–pancreas antigen) and serum tumor markers (AFP, CEA, CA125, CA19-9, CA15-3, CA72-4, FER, FPSA, PSA, SCC, NSE) were negative. No coagulopathy were observed. Iron and copper status were normal. Abdominal ultrasound revealed chronic diffuse hepatopathy, decreased size of gallbladder without gallstones or sludge and thicken gallbladder wall. There is no pericholecystic fluid. The common bile duct has a normal caliber. Enhanced Magnetic Resonance Imaging (MRI) and Magnetic Resonance CholangioPancreatography (MRCP, Figure 2) were normal.

**Figure 3:**
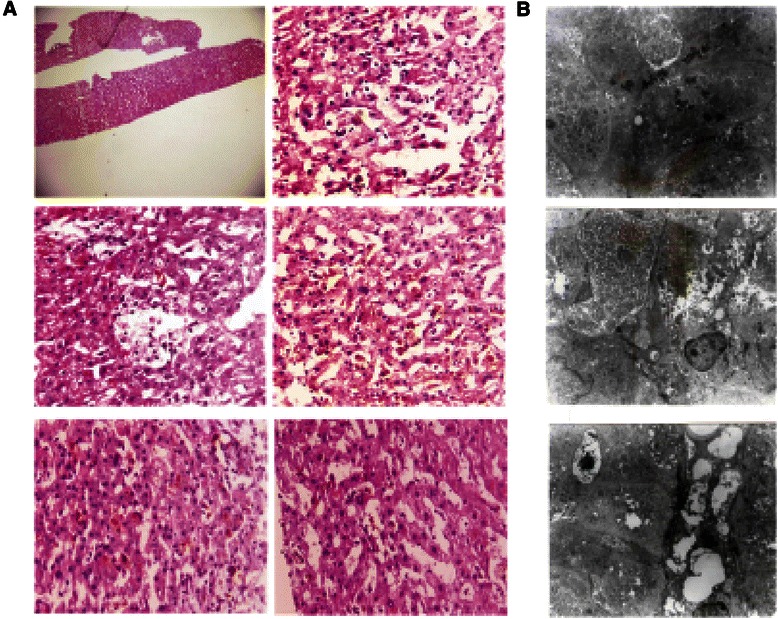
The representative images for Liver biopsy **(A)** and electron endoscopy**(B)** of this patient.

**Figure 4:**
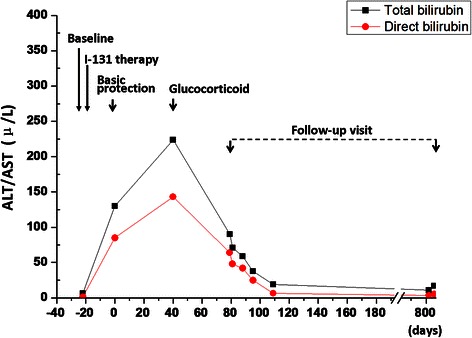
Line chart for the bilirubin level of this patient.

**Figure 5:**
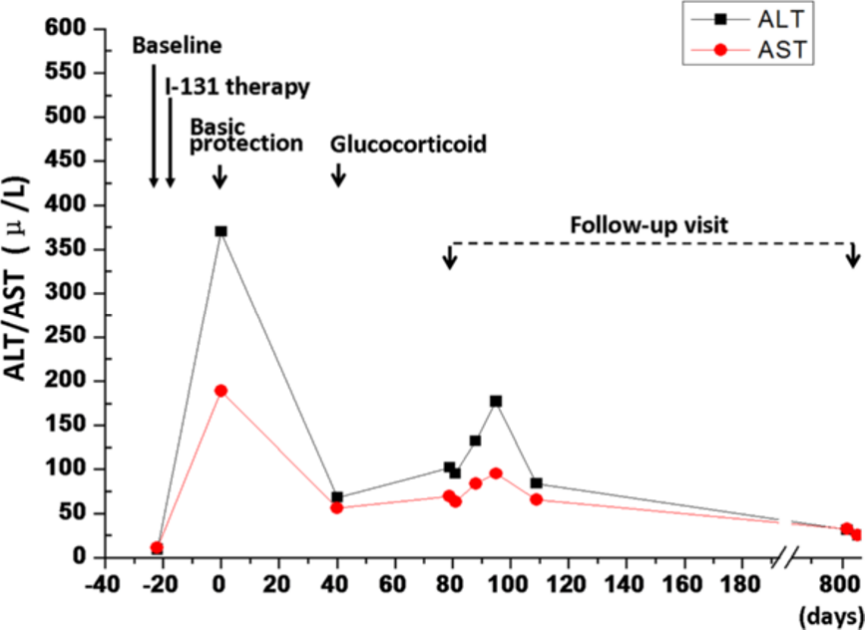
Line chart for the AST/ALT level of this patient.

The patient was initially treated with basic hepatic protection for 40 days, including glutathione, magnesium isoglycyrrhizinate, essential Nutrition, transmetil, and/or tauroursodeoxycholic acid. The total bilirubin was not efficiently controlled and kept increasing to 223.9 μmol/L 40 days later, even though the level of AST and ALT were decreased. Liver biopsy was further performed and the results showed swollen hepacytes, predominant piecemeal necrosis within the lobules, obvious canalicular and hepatocellular cholestasis [7]. The portal inflammation, hepatocyte rosettes and fibrosis were not detected (Figure 3). On the basis of Roussel Uclaf Causality Assessment Method (RUCAM = 6 for this patient, shown as Table 1) [8], it indicates the patient is probable I-131 administration induced liver injury. Therefore, the patient was further treated with methylprednisolone (40 mg iv drip qd*14d followed by maintenance and step-down withdrawal of oral methylprednisolone), the TB and ALT/AST were finally lowered back to normal in the end of follow-up visit (Figures 4 and 5). Considering the safety and validity to easterners, 40 mg iv drip qd is the standard initial dose in our institution for cholestatic DILI patients, who are insensitive to basis hepatic protection. The maintenance time and gradual reduction regime depend on the response of patients. Usually, the initial dose will be hold for the first 2 weeks, and the dose will be reduced 10% every 1-2 weeks when TB level was effectively controlled.

**Table 1:** **Causality assessment of this patient**

Criteria	Score
1. Time to onset of the reaction Suggestive	2 (5 to 90 days from the beginning of the initial I-131 treatment)
2. Course of the reaction	0 (no information )
3. Risk factor for drug reaction	1 (age of patient ≥55 years)
4. Concomitant drugs	0 (None)
5. Non drug-related causes	2 (None)
6. Previous information on the drug	1 (Reaction published but unlabelled)
7. Response to readministration	0 (Not done)
**Total**	**6**

## Discussion

Drug-induced liver injury(DILI) refers to liver injury caused by drugs or chemical agents, which represents viral hepatitis-like symptoms, malaise, anorexia, nausea and vomiting, right upper quadrant abdominal pain, jaundice, acholic stools, and dark urine, as a special type of adverse drug reaction [6,9,10]. DILI is one of the leading causes of acute liver failure in U.S.A., accounting for around 13% cases of acute liver failure [11]. In China, DILI causes 10% cases of acute hepatitis and 12.2% in acute liver failure. Exploring the risk of these events poses a major challenge for drug safety.

The therapeutic value of I-131 ablation therapy after total thyroidectomy has been used as an important component in thyroid cancer management [12,13]. Based on what is known so far, acute complications due to I-131 ablation therapy include transient parotitis and thyroiditis; reported chronic complications are amenorrhea, pulmonary fibrosis, azoospermia, aplastic anemia, and leukemia. The ablation-therapeutic I-131 induced DILI reaction has not been included either in I-131 product label or well-known reports.

Even though the radioactive I-131 becomes mainly toxic to thyroid cells that accumulate iodine from bloodstream, it also could be biodistributed to whole body and other organs. In total thyroidectomy patient, the lower gastrointestinal tract, kidney, stomach, heart wall and liver have been demonstrated to absorb relatively high doses per unit of administered iodine activity [14]. In this case, the emission computed tomography (ECT) I131 whole body scan demonstrated uptake mainly in the thyroid bed, but also in the liver, stomach, oral and nasal cavity 5 days after radioiodine treatment (Figure 1). Besides the biodistribution of I-131 in thyroidectomy patient, the high dose of I-131 used for ablation therapy might also be one of the reasons leading to DILI. Individual dose of I-131 ranges from 2 to 4 mCi for diagnosis, and ranges from 5 to 10 mCi for hyperthyroidism. However, it ranges from 75 to 150 mCi for therapeutic ablation therapy, which is 15 times higher than treating benign thyroid diseases and 35 times higher than monitoring purpose. Navina et al have reported the hepatotoxicity after I-131 treatment for 5 to 10 mCi each time in patients with Grave’s disease [15], which warns us to pay more attention to the possibility of hepatotoxicity induced by therapeutic I-131 ablation therapy with much higher I-131 dose.

## Conclusion

To our knowledge, there is rare description of DILI complications due to I-131 ablation therapy in thyroidectomy patient. The patient, who denies history of liver disease, presents typical cholestatic damage manifestation with short course of reaction to I-131 administration, and responds to glucocorticoid therapy well, but not basic hepatic protection treatment. This is only a case report from one patient data, but it holds the significance to reveal the possibility of ablation I-131 therapy induced liver injury. As an additional remarks, it reminds physicians make DILI in early consideration when patient present liver injury after I-131 ablation therapy.

### Consent

Written informed consent was obtained from the patient for publication of this case report and any accompanying images. A copy of the written consent is submitted to this journal.

## Abbreviations

DILI: Drug-induced liver injury
DHU: Diffuse hepatic uptake
I-131: Radioactive iodine
ECT: Emission Computed Tomography
AST: Aspartateaminotransferase
ALT: Alanin aminotransferase
TB: Direct bilirubin
GGT: Gamma-glutamyltransferase
ALP: Alkalinephosphatase
INR: International normalized ratio
MRI: Enhanced Magnetic Resonance Imaging
MRCP: Magnetic Resonance Cholangio Pancreatography
RUCAM: Roussel Uclaf Causality Assessment Method
